# Lead Is Toxic to Neuronal Cells by Inducing Oxidative Stress and Activating Neuroinflammatory Pathways

**DOI:** 10.3390/brainsci16080889

**Published:** 2026-08-20

**Authors:** Khulud Badawi, Abdulrahman Mujalli, Wadyan Owaydhah, Basma Elsharazly, Ping Chen, Vic K. T. Sun, Ola Negm, Raheela Khan, Wayne G. Carter

**Affiliations:** 1 Clinical Toxicology Research Group, School of Medicine, University of Nottingham, Royal Derby Hospital Centre, Uttoxeter Road, Derby DE22 3DT, UK; khulud.badawi1@nottingham.ac.uk (K.B.); wadyan.owaydhah@nottingham.ac.uk (W.O.); basma.elsharazly1@nottingham.ac.uk (B.E.); ping.chen@nottingham.edu.cn (P.C.); 2 Department of Clinical Laboratory Sciences, Faculty of Applied Medical Sciences, Umm Al-Qura University, Makkah 24382, Saudi Arabia; ammujalli@uqu.edu.sa; 3 Faculty of Pharmacy, Taibah University, Al Madinah Al Munawwarah 42353, Saudi Arabia; 4 Parasitology Department, Faculty of Medicine, Tanta University, Tanta 31527, Egypt; 5 Nottingham Ningbo China Beacons of Excellence Research and Innovation Institute, University of Nottingham Ningbo China, Ningbo 315153, China; 6 School of Medicine, University of Nottingham, Royal Derby Hospital Centre, Uttoxeter Road, Derby DE22 3DT, UK; vic.sun@nottingham.ac.uk (V.K.T.S.); ola.negm@nottingham.ac.uk (O.N.); raheela.khan@nottingham.ac.uk (R.K.)

**Keywords:** lead, neurotoxicity, neuroinflammation, transcriptomics, toxicogenomics

## Abstract

Background/Objectives: Exposure to lead (Pb) is a serious public health concern for which there is no safe level. The aim of this study was to investigate the toxicity of Pb to undifferentiated (uSH-SY5Y) and differentiated (dSH-SY5Y) human neuroblastoma cells and to evaluate gene transcription in response to sub-lethal lead exposure. Methods: Pb was applied to uSH-SY5Y and dSH-SY5Y cells across a concentration range of 0–5 mM for 4, 6, and 24 h, and cell viability was assessed using 3-(4, 5-dimethylthiazol-2-yl)-2, 5-diphenyltetrazolium bromide (MTT) and lactate dehydrogenase assays. Results: Pb induced a significant concentration- and exposure-dependent reduction in cell viability. Pb significantly impacted cellular bioenergetics and reduced ATP production in a concentration- and exposure duration-dependent manner, triggering elevated levels of deleterious reactive oxygen species. Transcriptomic profiling in dSH-SY5Y cells after a sub-lethal 24 h exposure to 1.25 mM Pb revealed 757 upregulated and 2206 downregulated genes. From Gene Ontology and KEGG pathway enrichment analysis, biological processes were predominantly associated with immune and inflammatory processes, including cytokine-mediated signalling. Upregulated differentially expressed genes (DEGs) included those for PI3K/Akt and cytokine signalling, and downregulated DEGs included genes linked to spinocerebellar ataxia, mitophagy, cytokine receptor interaction and cellular metabolism. Protein–protein interaction analysis identified six key hub-upregulated genes with a primarily inflammatory focus (CD44, CXCR4, PTGS2, IL1β, TNF, MMP9) and one downregulated gene (CD4) as potential regulators of Pb-induced cellular responses. Disease association analyses revealed links to chemical carcinogenesis and neurodegenerative diseases. Conclusions: Collectively, these findings provide molecular insights into Pb-induced neurotoxicity and highlight a network of genes that converge on neurological and inflammatory pathways, which are candidates for further mechanistic investigation and possible therapeutic targeting following Pb poisoning.

## 1. Introduction

Lead (Pb) is a persistent environmental pollutant and cumulative toxicant, with no recognised safe blood lead level (BLL) [1]. Lead exposure has been estimated to account for >1.5 million deaths globally in 2021 [1], with humans exposed through ingestion of foods and drinking water, as well as through inhalation and dermal routes, particularly in occupational settings [2]. Historically, lead exposure has typically arisen from anthropogenic sources such as mining and smelting, as well as the use of lead in paints, pottery, and gasoline [2,3]. Although Pb exposure has been in a progressive decline over the last 40 years [4], in part from the phased-out use of Pb in gasoline, Pb can bioaccumulate in humans [5]. Lead is primarily stored in bones and teeth, as well as other bodily tissues, including the liver, skeletal muscle, and brain [2,3,5,6]. Furthermore, bone can act as a reservoir for Pb, and with a half-life of 20 to 30 years, bone can release Pb back into the circulation, triggering chronic internal exposure.

Pb can damage cells and tissues, particularly those of the immune, haematopoietic, nervous, cardiovascular, renal, and reproductive systems [2,3,5,6], through the induction of protein and DNA damage, epigenetic changes, and mitochondrial dysfunction and oxidative stress [2,3,4,6,7,8]. Neurotoxic effects arise because Pb^2+^ can substitute for other divalent metal cations such as Ca^2+^ and cross the blood–brain barrier (BBB) [5,9]. Furthermore, Pb can accumulate in the brain with an estimated half-life of 2–3 years [5], potentially inducing chronic damage. Neurotoxic and poisoning effects reflect BLLs in adults, with symptoms ranging from possible hypertension and kidney dysfunction at a BLL of 5–10 μg/dL (0.24–0.48 µM), cognitive impairment at 40–79 μg/dL (1.9–3.8 µM), neuropathy at >80 μg/dL, and encephalopathy at 100–120 μg/dL (4.8–5.8 µM) [2,10]. Notably, Pb exposure is a risk factor for preterm birth, and exposure during pregnancy or early childhood can have a profound effect on neurodevelopment; higher BLLs correlate with reduced IQ in children [2,4], and childhood Pb exposure and age-adjusted BLL are risk factors for attention-deficit/hyperactivity disorder (ADHD) [11]. To identify children with relatively high blood Pb levels and mitigate risk, the US Centers for Disease Control and Prevention (CDC) reduced the blood lead reference value (BLRV) from 5.0 μg/dL to 3.5 μg/dL in 2021 [12].

Neurotoxic mechanisms of Pb have been studied in bulk tissue, cell subtypes, as well as single cells [13]. Pb exposure can induce cellular redox stress in brain tissues and disrupt cellular homeostasis during neurodevelopment (vide supra) and during adulthood, triggering cell damage and increased markers of neurodegeneration [13,14].

Cultured and primary cells provide a useful means to study the neurotoxic effects of Pb. SH-SY5Y cells are a widely utilised model for neurotoxicity and can be differentiated into neuronal cells with a catecholaminergic phenotype [15]. Studies with SH-SY5Y cells have provided insight into Pb neurotoxicity [16,17]. However, the acute concentration–toxicity relationship with Pb and associated transcriptomic responses has not been defined in this cellular model. In addition, a direct comparison of undifferentiated (uSH-SY5Y cells) with their differentiated counterparts with respect to acute toxicity thresholds has not been conducted. These knowledge gaps are important because differentiation triggers cell cycle arrest, producing non-proliferating neurons, neuritic arborisation, and neuronal marker expression [15,18], all of which could influence susceptibility to Pb toxicity. Therefore, our hypothesis to test is that the differentiation of SH-SY5Y cells into neurons would shift their acute toxicity profile (to Pb) and Pb would induce a stress-related early transcriptomic response. To address these research gaps, we assessed Pb toxicity in uSH-SY5Y and differentiated (dSH-SY5Y) cells, applied transcriptomic profiling (RNA sequencing) to identify differentially expressed genes (DEGs) induced by Pb exposure, and mapped their interactions using comprehensive bioinformatic analyses. The identification of DEGs may highlight potential markers of Pb-induced toxicity and targets for mechanistic and therapeutic investigation.

## 2. Materials and Methods

All materials were purchased from Sigma (Poole, UK), unless specified otherwise.

### 2.1. Cell Culture and Lead Treatment

SH-SY5Y neuroblastoma cells, originally purchased from the European Collection of Authenticated Cell Culture (ECACC) (ECACC-94030304), were used to evaluate the cytotoxic effects of Pb. SH-SY5Y cells (passage #15) were seeded in T25 flasks (ThermoFisher Scientific, Rochester, UK) at a density of 3 × 10^4^ cells/well in a medium of 43.5% Eagle’s minimum essential medium (EMEM), 43.5% Ham’s F12 nutrient mix, 1% MEM non-essential amino acid solution (NEAA), 2 mM glutamine, 10% heat-inactivated foetal bovine serum (FBS) (F9665, Sigma, Poole, UK), and 1% penicillin–streptomycin solution (containing 10 mg/mL streptomycin and 10,000 IU/mL penicillin (P4333, Sigma, Poole, UK)) at 37 °C with an atmosphere of 5% CO_2_ and 95% humidity as described previously [19]. For differentiation, SH-SY5Y cells were differentiated in a medium of 48% EMEM, 48% Ham’s F12 nutrient mix, 1% NEAA, 2 mM glutamine, 1% heat-inactivated FBS, and 1% penicillin–streptomycin solution containing 10 µM all-trans retinoic acid (RA) (Millipore, Burlington, MA, USA), as previously described [19,20]. Cells were treated with RA typically up to 10–11 days and/or until they reached approximately 90% confluency (Supplementary Material). dSH-SY5Y displayed neurite projections and had an increased expression of neuronal markers (Supplementary Material Figure S1). Lead (II) nitrate (Pb^2+^ (NO_3_)^2−^) (henceforth referred to as Pb) was applied to SH-SY5Y cells (80–85% confluent) in the culture medium at final concentrations of 0.3125, 0.625, 1.25, 2.5, and 5 mM (10,350–165,600 µg/dL), for up to 24 h. Cell samples were tested using a PlasmoTest™—Mycoplasma Detection Kit (Invivogen, Toulouse, France) and were confirmed negative for mycoplasma.

### 2.2. Assessment of Lead Cytotoxicity by Cell Viability Assays

After incubation with Pb at 37 °C (concentrations of 0.3125–5 mM for 4–24 h), the cell culture medium was removed and replaced with new growth medium containing 0.5% (*w*/*v*) 3-(4,5-dimethylthiazol-2-yl)-2,5-diphenyltetrazolium bromide (MTT), and MTT cell metabolic assays performed as described previously [20], with absorbance readings at 570 nm using a Varioskan™ LUX multimode microplate reader (Thermo Electron Corporation, Waltham, MA, USA). Lactate dehydrogenase (LDH) assays were undertaken using a CyQUANT, LDH cytotoxicity assay kit (Thermo Fisher Scientific, Waltham, MA, USA) according to the manufacturer’s guidelines, as detailed in a previous publication [20]. The final product was measured spectrophotometrically at 490 nm and 680 nm using a Varioskan™ LUX multimode microplate reader (Thermo Fisher Scientific, Waltham, MA, USA). The absorbance value at 680 nm was subtracted from the absorbance value at 490 nm to calculate LDH activity, as previously described [20]. The OD values from each treatment were normalised to the mean of the negative control, and the percentage of LDH production was determined. Intracellular ATP levels were quantified using the ATP Bioluminescence Assay Kit CLS II (product 11 699 695 001, Roche, Mannheim, Germany). Cells were grown in 6-well plates and treated with Pb as described above, and ATP levels were quantified by luminescence using a Varioskan™ LUX multimode microplate reader (Thermo Fisher Scientific, Waltham, MA, USA) and interpolation from a standard curve, as previously described [20]. For cell viability assays, the viability of vehicle control-treated cells was set to 100%.

### 2.3. Analysis of Reactive Oxygen Species Induced by Lead

The quantification of the level of reactive oxygen species (ROS) in cell extracts was conducted using a 2′,7′-dichlorofluorescein diacetate (DCFDA) assay. Cells were plated in 96-well clear-bottomed plates (165305, Thermo Fisher Scientific, Rochester, UK) and then treated with Pb (at concentrations of 0.3125–5 mM for 24 h), as described above. Hydrogen peroxide (H_2_O_2_) was used as positive control for the generation of ROS and applied at 500 µM for 30 min. DCFDA (50 µM) was introduced to each well 30 min prior to the conclusion of the experiment, allowing time for cellular integration. After the treatments, cells were washed twice with ice-cold phosphate-buffered saline (PBS), and fluorescence levels were quantified as described previously [21].

### 2.4. RNA Extraction and Transcriptome Sequencing

Differentiated SH-SY5Y cells were prepared as described above and then treated with or without Pb at a concentration of 1.25 mM for 24 h. RNA was extracted using the RNeasy Mini Kit (Qiagen, Manchester, UK) following the manufacturer’s instructions. RNA was eluted in 30 μL of nuclease-free water, and the concentration of eluted RNA was determined using a VarioSkan LUX spectrophotometer (Thermo Fisher Scientific, Waltham, MA, USA), coupled with a µDrop plate, and then immediately stored at −80 °C. RNA purity was determined using an A260:A280 ratio, and RNA integrity was assessed by gel electrophoresis. All samples were confirmed to be of high purity (results not included) and were used for subsequent transcriptomic analysis.

Transcriptome sequencing was conducted by Novogene (https://www.novogene.com) using the NovaSeq X Plus platform. Raw sequencing reads were subjected to quality control using fastp to remove adapter sequences, poly-N reads, and low-quality reads. High-quality clean reads were aligned to the human reference genome using HISAT2 (v2.0.5). RNA-seq quality metrics were assessed following read alignment. Principal component analysis (PCA) was performed to evaluate sample clustering and identify potential outliers. Data visualisation was performed in R (v4.5.0) using ggplot2 package. Gene expression levels were quantified using featureCounts (v1.5.0), and transcript abundance was estimated as fragments per kilobase of transcript per million mapped reads (FPKM). Differential gene expression between Pb-treated dSH-SY5Y and control cells was analysed using the DESeq2 R package (v1.20.0). Differential expression was defined by an absolute log_2_ fold change (|log_2_FC| ≥ 1) and an adjusted *p*-value < 0.05 based on the Benjamini–Hochberg correction for multiple testing [22]. Genes exhibiting a log_2_FC > 1 were considered upregulated genes, and those with a log_2_FC < −1 were considered downregulated genes in Pb-treated cells relative to controls. DEGs were subjected to K-means clustering analysis based on normalised expression profiles. A cluster-specific functional enrichment analysis was subsequently performed using the clusterProfiler package.

### 2.5. Functional Enrichment Analysis

Differentially expressed genes (DEGs) underwent functional enrichment analysis to identify enriched biological processes, molecular functions, cellular components, and pathways. The DEGs were converted to Entrez Gene IDs using the org.Hs.eg.db annotation database [22,23]. Gene Ontology (GO) enrichment analysis was performed utilising the clusterProfiler package, which facilitates over-representation analysis within the three GO domains: biological process (BP), molecular function (MF), and cellular component (CC). Kyoto Encyclopedia of Genes and Genomes (KEGG) pathway enrichment analysis was performed using the enrichKEGG function in clusterProfiler. Data processing and result summarisation were conducted using dplyr, while visualisations of enriched GO terms and KEGG pathways were generated with the ggplot2 package [24]. All analyses were performed in R (v4.5.0).

### 2.6. Protein–Protein Interaction Network and Hub Gene Analysis

Protein–protein interaction (PPI) networks of DEGs were constructed using the STRING database (https://string-db.org/) with a confidence score cut-off of 0.4 and visualised using Cytoscape (v3.10.3). Functional modules within the PPI network were identified using the Molecular Complex Detection (MCODE) plugin with default parameters. Hub genes were ranked using the CytoHubba plugin based on degree, maximal clique centrality (MCC), and bottleneck algorithms. Genes consistently identified across the three ranking methods were considered candidate hub genes.

### 2.7. Disease Association Analysis

To investigate the clinical relevance of the selected feature genes, gene–disease association analyses were performed using DisGeNET (http://www.disgenet.org) and the Comparative Toxicogenomics Database (CTD) (https://ctdbase.org/). Hub genes were queried to retrieve curated disease associations, with a particular focus on neurological and nervous system-related disorders. The resulting networks were visualised using Cytoscape (v3.10.3).

### 2.8. Statistical Analysis

All data sets were derived from at least three distinct studies and expressed as the mean ± standard error of the mean (SEM). GraphPad Prism 10 (GraphPad Prism, San Diego, CA, USA) was used for the data analysis. A nonlinear regression curve fit model was used to calculate IC_50_ values, while a dose–response curve was used to determine EC_50_. To compare the control and treatment groups, analysis of variance (ANOVA) was employed, using either two-way (Dunnett’s multiple comparisons test) or one-way (Tukey’s multiple comparisons test) analyses. Pearson’s correlation analysis was also conducted.

## 3. Results

### 3.1. Pb Is Toxic to SH-SY5Y Cells and Induces a Loss of Cell Viability

**Figure 1:**
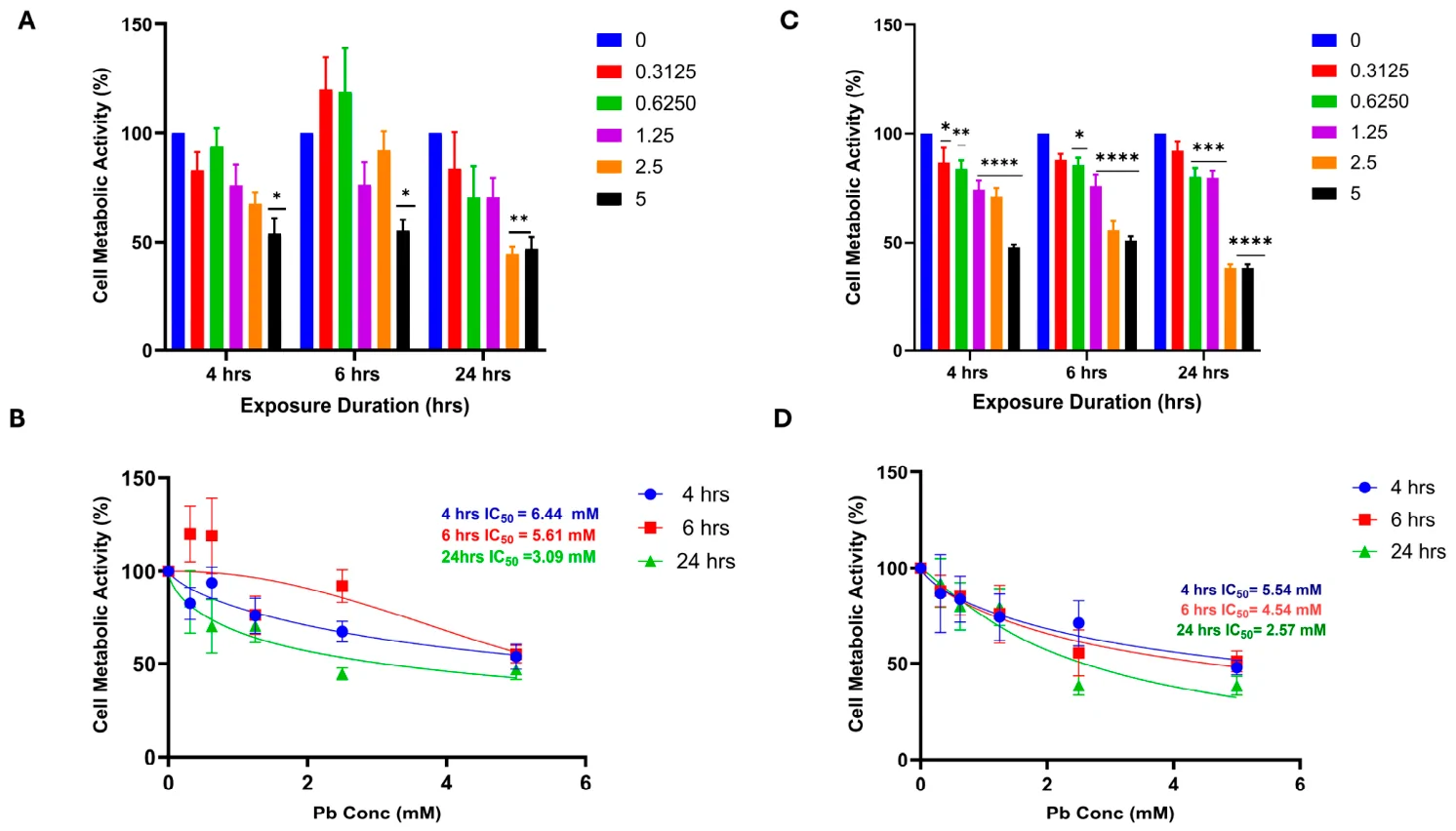
Pb effects on the cell viability of uSH-SY5Y and dSH-SY5Y cells analysed by an MTT assay. uSH-SY5Y and dSH-SY5Y cells were treated with Pb at concentrations of 0.3125–5 mM for 4, 6, and 24 h. Cell metabolic activity was assessed using an MTT assay. Absorbance readings were normalised to the viability of vehicle controls, providing cell viability as a percentage relative to the vehicle control. Readings were taken from three independent experiments with three technical replicates per concentration (*n* = 3). The histograms in panels (**A**) (uSH-SY5Y) and (**C**) (dSH-SY5Y) show the time- and concentration-dependent reduction in cell viability. Statistical analysis was performed using two-way ANOVA followed by Dunnett’s multiple comparisons test. Data are presented as mean ± SEM. For significance levels: * *p* < 0.05, ** *p* < 0.01, *** *p* < 0.001, **** *p* < 0.0001 relative to control values set at 100%. Panels (**B**) (uSH-SY5Y) and (**D**) (dSH-SY5Y) show nonlinear regression curves generated using a four-parameter variable slope inhibitory dose–response model to estimate the IC_50_ values at each exposure duration.

Lead exposure to undifferentiated and differentiated (dSH-SY5Y) cells produced a concentration- and exposure duration-dependent decrease in neuronal cell viability, as evidenced by an MTT assay (Figure 1A–D). Differentiated cells were more vulnerable to the toxic effects of Pb, with lower estimated IC_50_ values (Figure 1A–D and refer to Table 1).

**Figure 2:**
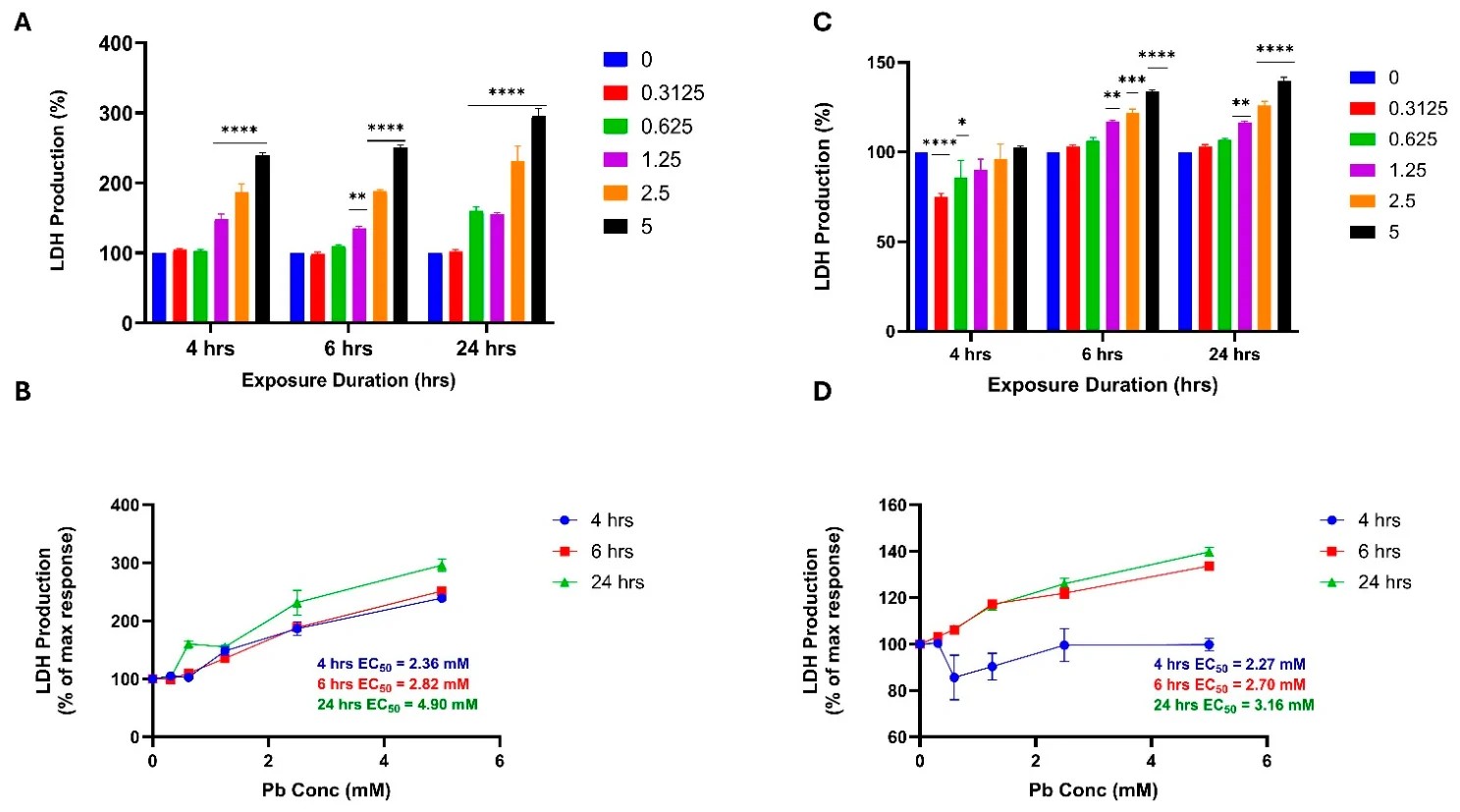
Pb effects on the cell viability of undifferentiated and dSH-SY5Y cells analysed by an LDH assay. uSH-SY5Y and dSH-SY5Y cells were treated with Pb at concentrations of 0.3125–5 mM for 4, 6, and 24 h, and cell viability was quantified using an LDH activity assay. Absorbance readings were obtained from three independent experiments, each with three technical replicates per condition (*n* = 3). The histograms in panels (**A**) (uSH-SY5Y) and (**C**) (dSH-SY5Y) show the time- and concentration-dependent increases in LDH release. Statistical analysis was performed using two-way ANOVA followed by Dunnett’s multiple comparisons test. Data are presented as mean ± SEM. Significance levels: * *p* < 0.05, ** *p* < 0.01, *** *p* < 0.001, **** *p* < 0. 0001 relative to control values set at 100%. Panels (**B**) (uSH-SY5Y) and (**D**) (dSH-SY5Y show nonlinear regression curves generated using the [Agonist] vs. response–variable slope model in GraphPad Prism to estimate the EC_50_ values at each exposure duration.

Similarly, there was a significant elevation of extracellular LDH activity, as a marker of reduced cell viability, that was proportional to Pb concentration and exposure duration for uSH-SY5Y and dSH-SY-5Y cells (Figure 2A–D). As with the MTT assay, the estimated half-maximal effective concentration (EC_50_ values) was lower in dSH-SY-5Y cells, indicating greater vulnerability to Pb toxicity following cellular differentiation, although with less total LDH produced (refer to Table 1).

**Figure 3:**
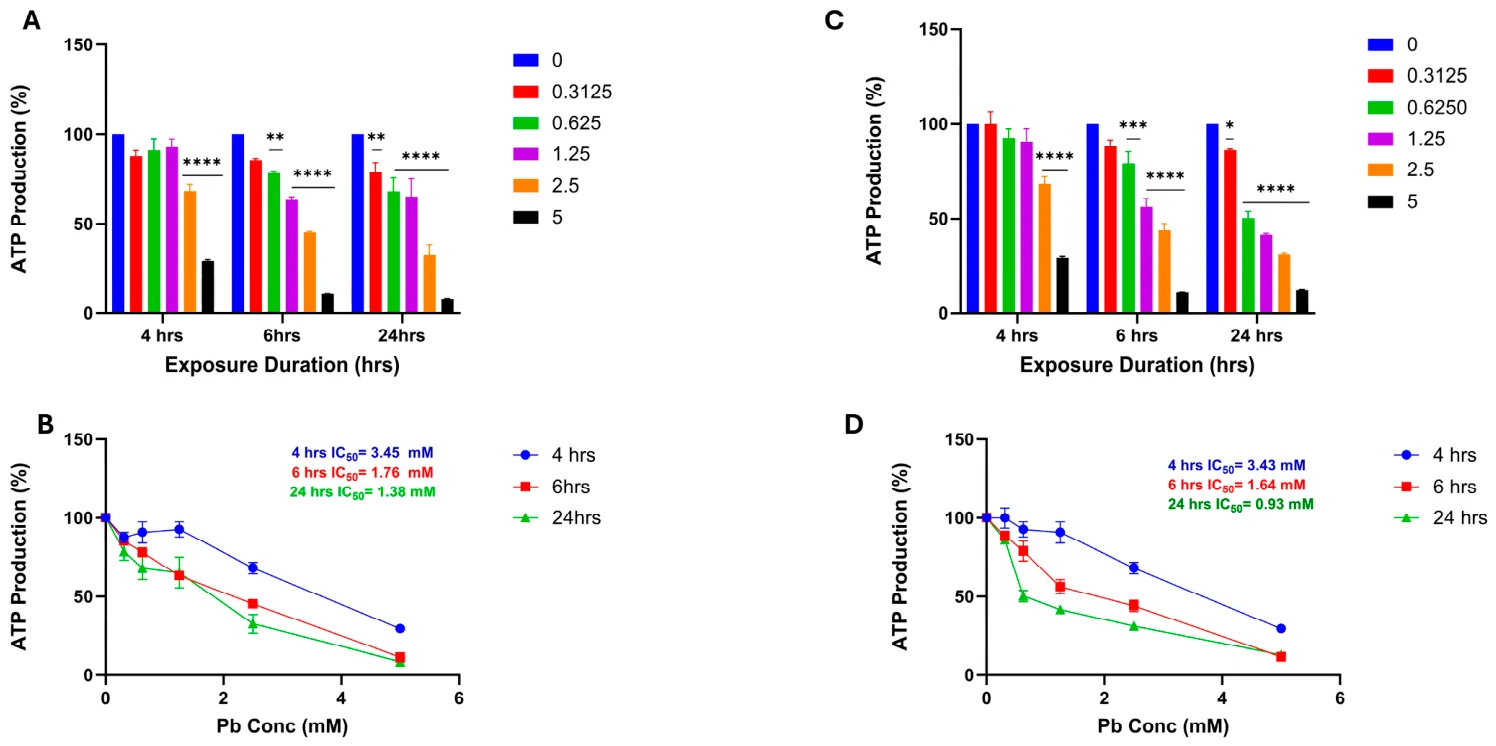
Pb effects on the cellular bioenergetics of uSH-SY5Y and dSH-SY5Y cells analysed by an ATP assay. uSH-SY5Y and dSH-SY5Y cells were treated with Pb at concentrations of 0.3125–5 mM for 4, 6, and 24 h, and intracellular ATP levels were measured using a luminescence-based assay. Luminescence readings were first corrected by subtracting background signal from blank wells, then normalised to the intensity of vehicle-treated controls, providing ATP levels as a percentage relative to untreated baseline values. Data were collected from three independent experiments, each with three technical replicates per condition (*n* = 3). The histograms in panels (**A**) (uSH-SY5Y) and (**C**) (dSH-SY5Y) show Pb-induced changes in ATP levels across time points and concentrations. Statistical analysis was performed using two-way ANOVA followed by Dunnett’s multiple comparisons test. Data are presented as mean ± SEM. For significance levels: * *p* < 0.05, ** *p* < 0.01, *** *p* < 0.001, **** *p* < 0. 0001 relative to control values set at 100%. Panels (**B**) (uSH-SY5Y) and (**D**) (dSH-SY5Y) show nonlinear regression curves generated using the [Inhibitor] vs. normalised response–variable slope model in GraphPad Prism to estimate the IC_50_ values at each exposure duration.

Treatment of uSH-SY5Y and dSH-SY5Y cells with Pb resulted in damage to the cellular bioenergetic capacity and a corresponding concentration- and duration-dependent reduction in cellular ATP production (Figure 3A–D). As with the other cell viability assays, dSH-SY5Y cells were more susceptible to Pb as a toxic insult, with lower approximate IC_50_ values (see Table 1). Furthermore, cellular bioenergetic activity (ATP production) for uSH-SY5Y or dSH-SY5Y cells was more sensitive than the other cell viability assays to Pb exposures, with lower estimated IC_50_ values.

### 3.2. Pb Induces Production of Reactive Oxygen Species

**Figure 4:**
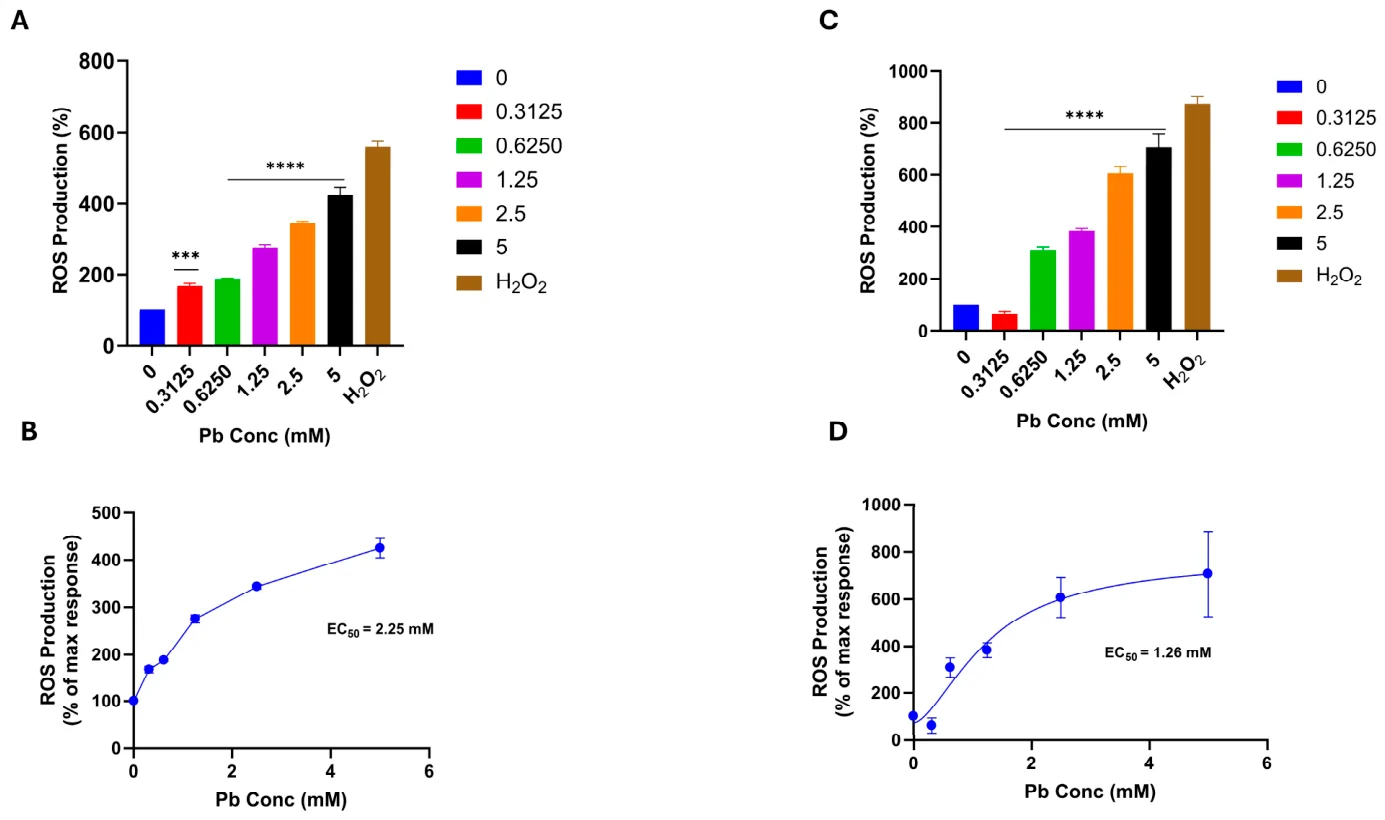
Pb induction of cellular ROS in uSH-SY5Y and dSH-SY5Y cells quantified using a DCFDA assay. uSH-SY5Y and dSH-SY5Y cells were treated with Pb at concentrations of 0.3125–5 mM for 24 h, and cellular reactive oxygen species (ROS) levels were measured using a fluorescent DCFDA assay. Fluorescence readings were first corrected by subtracting blank values, then normalised to the fluorescence intensity of vehicle-treated controls, providing ROS levels as a percentage relative to untreated baseline values. Hydrogen peroxide (H_2_O_2_) was used as a positive control for ROS induction. Readings were obtained from three independent experiments, each with three technical replicates per condition (*n* = 3). The histograms in panel (**A**) (uSH-SY5Y) and (**C**) (dSH-SY5Y) display ROS levels induced by Pb. Statistical analysis was performed using one-way ANOVA followed by Dunnett’s multiple comparisons test. Data are presented as mean ± SEM. Significance levels: *** *p* < 0.001, **** *p* < 0.0001 relative to control values. Panels (**B**) (uSH-SY5Y) and (**D**) (dSH-SY5Y) show nonlinear regression curves generated using the [Agonist] vs. response–variable slope model in GraphPad Prism to estimate the EC_50_ values.

Damaged mitochondria can trigger enhanced release of deleterious ROS. Hence, the effect of Pb exposure on cellular ROS production was examined using a DCFDA assay. Incubation of uSH-SY5Y and dSH-SY5Y cells with 0.3125–5 mM Pb induced a concentration-dependent increase in ROS production that was higher in dSH-SY5Y cells (Figure 4A–D).

### 3.3. Pb Induces Changes to the Transcriptome in dSH-SY5Y Cells

**Figure 5:**
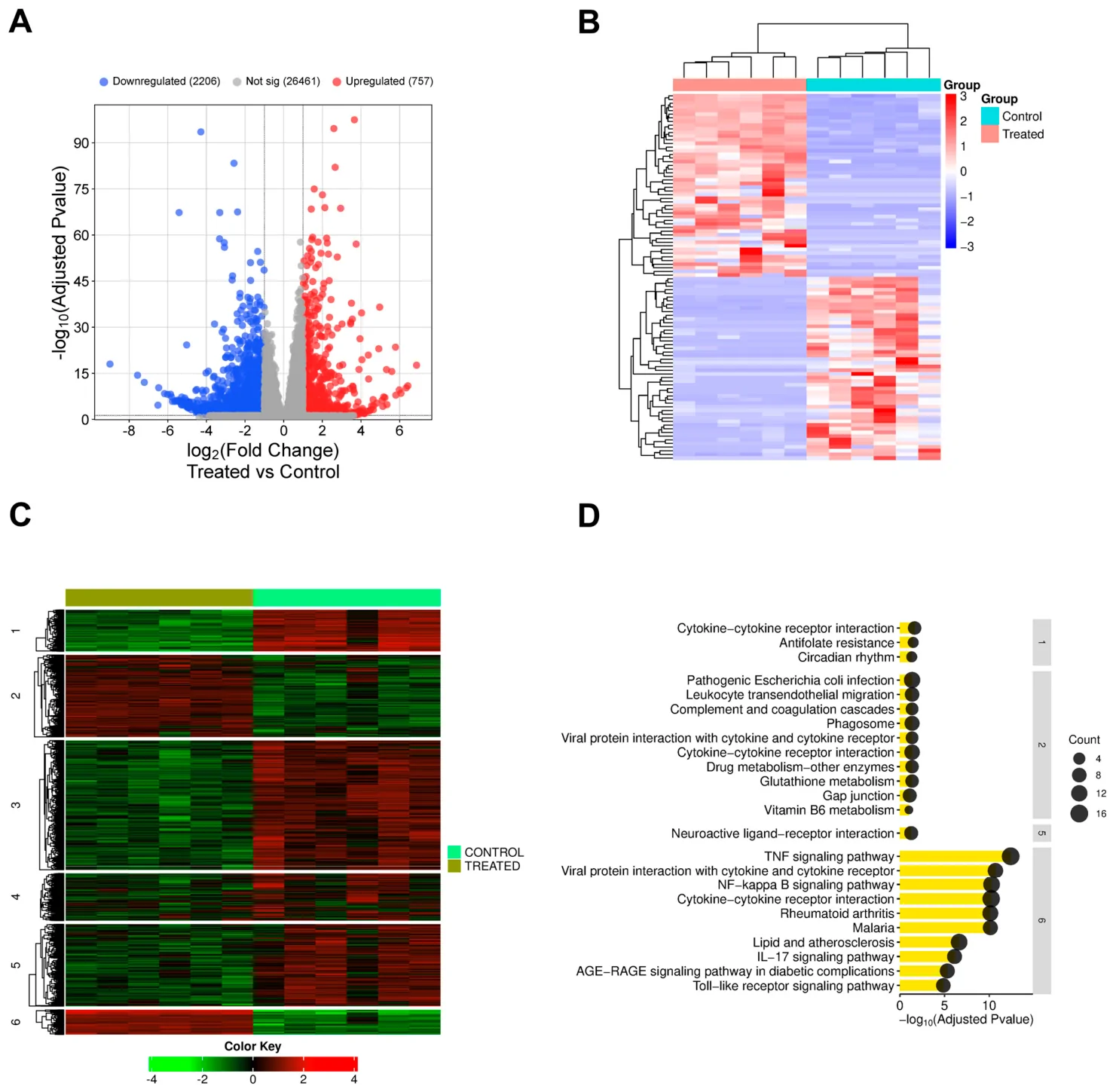
Transcriptomic profiling in dSH-SY5Y cells treated with Pb. dSHSY-5Y cells were treated with Pb at 1.25 mM for 24 h and transcriptomic profiling was performed. (**A**) Volcano plot showing the distribution of differentially expressed genes (DEGs) between Pb-treated and control cells. Upregulated genes (*n* = 757) are marked in red, downregulated genes (*n* = 2206) in blue, and non-significant genes (*n* = 26,461) in grey (adjusted *p* < 0.05, |log_2_FC| ≥ 1). (**B**) Heatmap of hierarchical clustering that illustrates distinct gene expression patterns between treated and control groups, with red indicating upregulation and blue indicating downregulation. (**C**) K-means clustering of DEGs that identifies six gene clusters based on expression patterns in Pb-treated and control cells. (**D**) KEGG enrichment of top cluster-specific genes; dot size indicates gene count, and colour reflects –log_10_ adjusted *p*-value.

From the cell viability assays (Figure 1, Figure 2 and Figure 3), exposure to 1.25 mM Pb for 24 h in dSH-SY5Y cells will induce cell damage and some cell death, indicating a toxic concentration of Pb but below the threshold for total cell loss. Hence, exposure to 1.25 mM Pb to dSH-SY5Y cells was chosen for transcriptomic profiling. RNA from six independent control experiments and six independent Pb-treated experiments was prepared, and mRNA sequencing was undertaken. The workflow employed in the RNA sequencing (RNAseq) project, along with the bioinformatic analysis pipeline used to process the raw data, is presented in the Supplementary Material (Figure S2). The RNAseq raw data were submitted to the Gene Expression Omnibus (GEO) repository (https://www.ncbi.nlm.nih.gov/geo/, and have been assigned the GEO accession number GSE343164. Exploratory data analysis and sample clustering of RNA-seq profiles were performed to assess sample variation and gene clustering, revealing clear separation between genes from control and Pb-treated cells, indicative of distinct transcriptomic profiles (Supplementary Material Figure S3). Although many genes were commonly expressed, their expression levels differed significantly, indicating differential transcriptional regulation in response to Pb exposure (Supplementary Material Figure S3). Differentially expressed gene (DEG) analyses were conducted to identify differences in gene expression profiles between Pb-treated and control cells (Figure 5A). A total of 2963 DEGs were identified, of which 757 genes were significantly upregulated, and 2206 genes were downregulated, while 26,461 genes showed no significant change (Figure 5A). Subsequently, a heatmap of DEGs was created to show the expression profiles of Pb-treated and control cells, resulting in hierarchical clusters (Figure 5B). To further explore expression patterns in response to Pb exposure, DEGs were grouped using k-means clustering. Six distinct gene clusters were identified, and a top cluster-specific pathway analysis revealed enrichment in elements, such as cytokine–cytokine receptor interaction, neuroactive ligand–receptor interaction, and signalling via TNF, NF-κB, AGE-RAGE, IL-17, and Toll-like receptors (Figure 5C,D).

**Figure 6:**
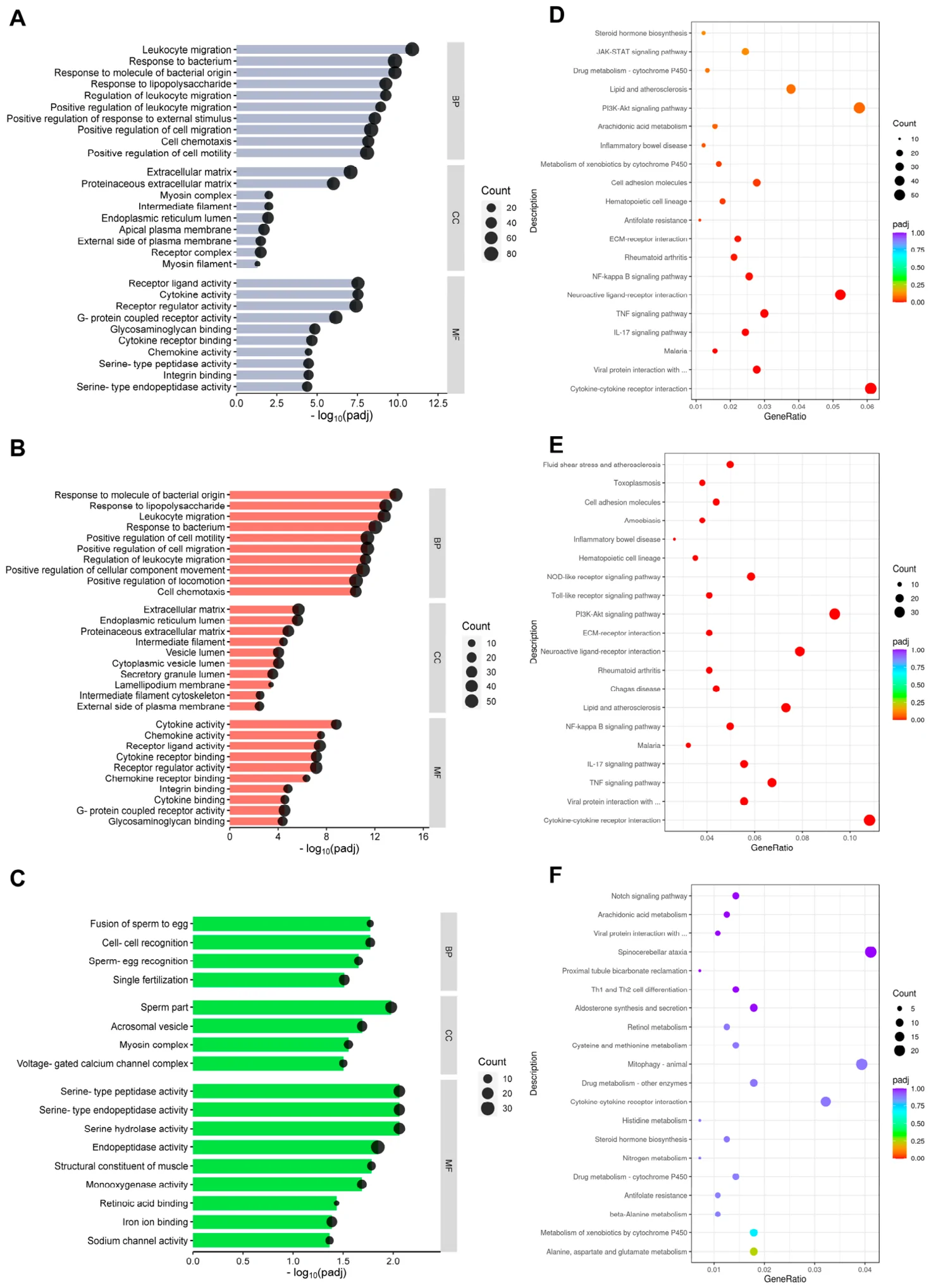
Functional annotation and pathway enrichment analysis of DEGs in dSHSY-5Y cells treated with Pb. dSHSY-5Y cells were treated with Pb at 1.25 mM for 24 h and transcriptomic profiling was performed. Bar plots represent GO enrichment analysis for (**A**) all DEGs, (**B**) upregulated DEGs, (**C**) downregulated DEGs, categorised into biological process (BP), cellular component (CC), and molecular function (MF). Dot plots show KEGG pathway enrichment analysis for all DEGs (**D**), upregulated DEGs (**E**) and downregulated DEGs (**F**). Dot size represents the number of genes per pathway, while dot colour reflects statistical significance via an adjusted *p*-value.

To gain insight into the biological significance of the transcriptomic changes induced by Pb exposure, GO and KEGG pathway enrichment analyses were performed on all DEGs (Figure 6A) and the DEGs were stratified into upregulated (Figure 6B) and downregulated (Figure 6C) subsets. GO enrichment analysis revealed that biological processes (BP) were predominantly associated with immune and inflammatory responses, including leukocyte migration, cytokine-mediated signalling, and response to bacterial molecules. Enriched cellular components (CCs) included the extracellular matrix, endoplasmic reticulum lumen, and plasma membrane. Molecular functions (MFs) were significantly enriched for cytokine and chemokine activity, and cytokine and G-protein coupled receptor binding (Figure 6A,B). KEGG pathway analysis also revealed that DEGs were significantly enriched in inflammation- and immune-related signalling pathways. Key enriched pathways across all DEGs included PI3K-AKT, NF-κB, IL-17, TNF signalling, and cytokine–cytokine receptor interactions (Figure 6D). Upregulated DEGs were particularly enriched in PI3K-AKT and cytokine signalling pathways (Figure 6E). Downregulated DEGs were enriched in pathways related to spinocerebellar ataxia, mitophagy, cytokine receptor interaction, cellular metabolism, neurodevelopment, and homeostasis (Figure 6F).

**Figure 7:**
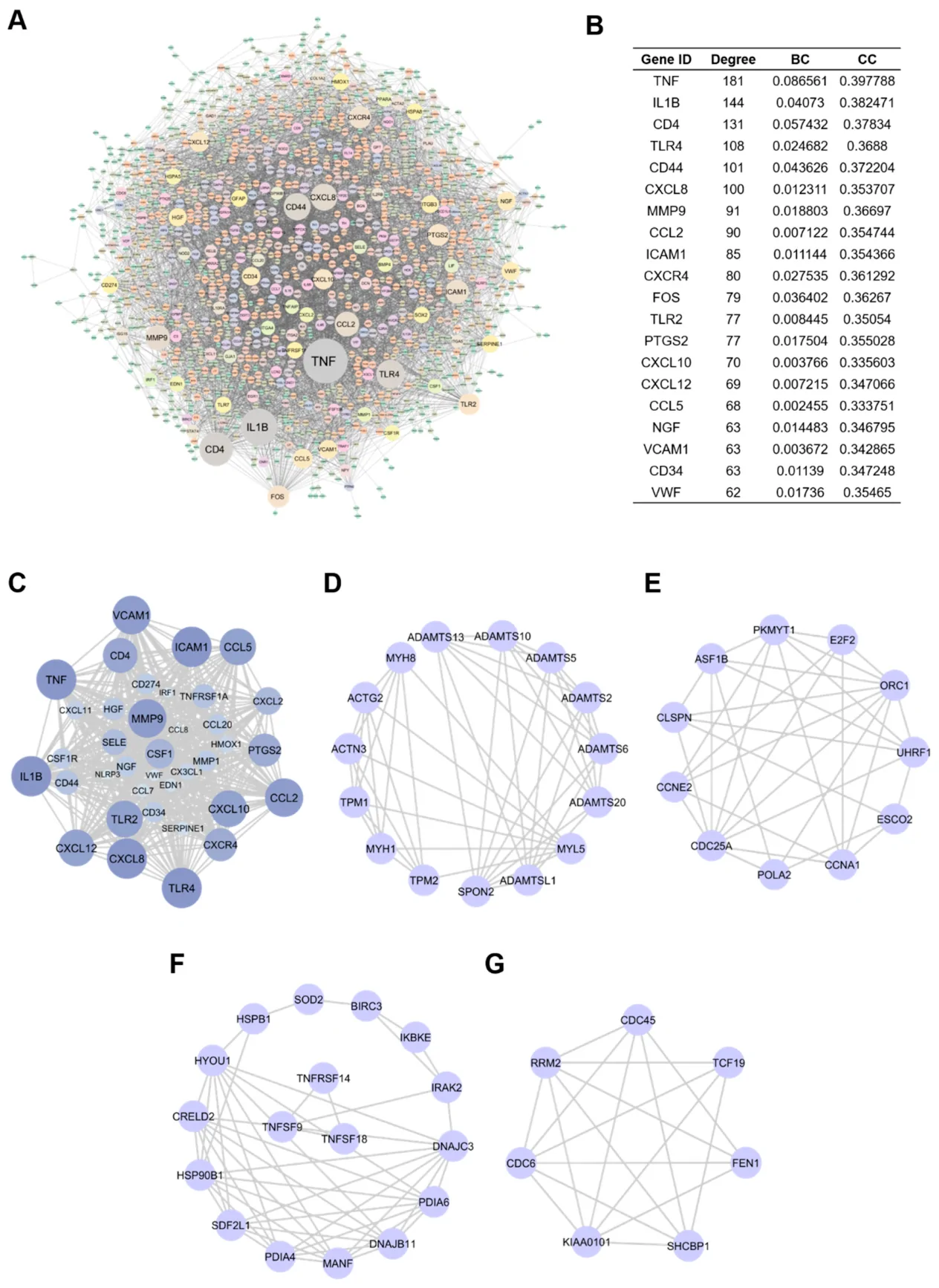
PPI network and module analysis of DEGs in dSHSY-5Y cells treated with Pb. dSHSY-5Y cells were treated with Pb at 1.25 mM for 24 h and transcriptomic profiling was performed. (**A**) Global PPI network constructed using the STRING database, visualised in Cytoscape. (**B**) Top genes ranked by degree centrality, with node size indicating the number of direct connections (degree). (**C**–**G**) MCODE-identified functional modules with MCODE scores ≥ 6.

To explore the interaction landscape of DEGs affected by Pb exposure, a protein–protein interaction (PPI) network was constructed. The PPI network revealed dense interconnections among the DEGs (Figure 7A). Topological analysis based on degree centrality, bottleneck centrality (BC), and closeness centrality (CC) identified several highly connected nodes, including TNF, IL1β, CD4, TLR4, CD44, CXCL8, MMP9, CCL2, ICAM1, and CXCR4 as potential central regulators (Figure 7B). The MCODE plugin was used to identify functionally significant subnetworks, extracting five highly interconnected modules with MCODE scores of ≥6 (Figure 7C–G). Module 1 with MCODE score of 29 (nodes = 37, edges = 522), module 2 with MCODE score of 7.14 (nodes = 15, edges = 50), module 3 with MCODE score of 6.6 (nodes = 11, edges = 33), modules 4 with MCODE score of 6 (nodes = 17, edges = 48) and modules 5 with MCODE score of 6 (nodes = 7, edges = 18) (Figure 7C–G).

**Figure 8:**
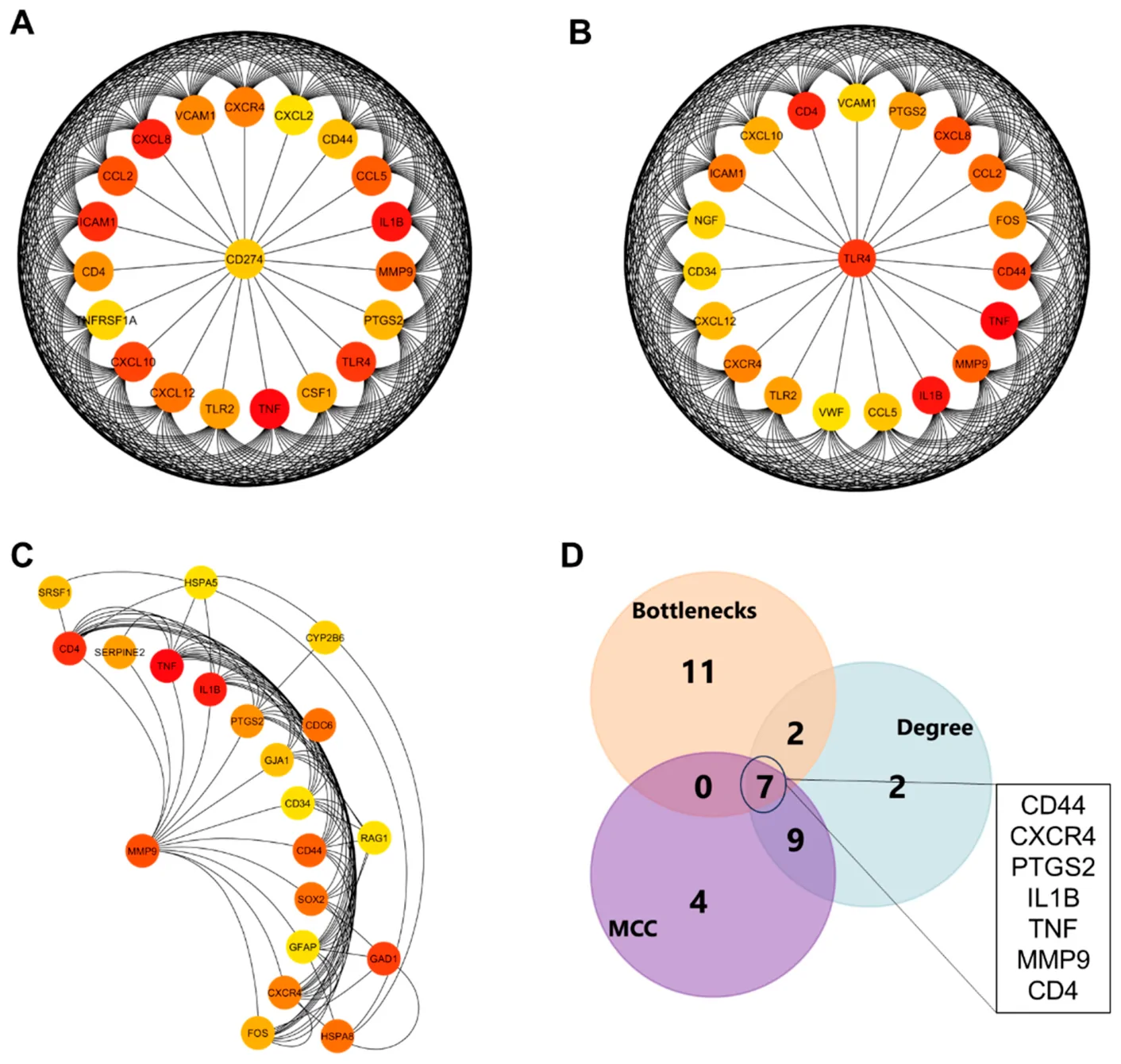
Identification of hub and bottleneck genes in the PPI network in dSHSY-5Y cells treated with Pb. dSHSY-5Y cells were treated with Pb at 1.25 mM for 24 h and transcriptomic profiling was performed. (**A**–**C**) Top 20 genes ranked by maximal clique centrality, degree, and bottleneck algorithms using the CytoHubba plugin in Cytoscape. Node colour intensity reflects rank (red = highest). (**D**) Venn diagram showing the overlap of top-ranked genes across the three methods, identifying seven genes (CD44, CXCR4, PTGS2, IL1B, TNF, MMP9, and CD4).

To prioritise hub genes within the PPI network, a topological ranking was performed using the CytoHubba plugin with three centrality measures: maximal clique centrality (MCC), degree, and bottleneck. The top 20 ranked genes for each method were determined (Figure 8A–C), with method-specific candidate genes identified. MCC analysis uniquely highlighted CD274, TNFRSF1A, CXCL2, and CSF1 as top-ranking genes, while NGF and VWF were uniquely identified via degree centrality. The bottleneck centrality revealed eleven unique genes: RAG1, HSPA8, SERPINE2, CDC6, HSPA5, CYP2B7P, GFAP, GAD1, SOX2, GJA1, and SRSF1. Comparative analysis across all three ranking methods revealed seven consistently overlapping hub genes: six upregulated (CD44, CXCR4, PTGS2, IL1β, TNF, and MMP9) and one downregulated (CD4) (Figure 8D), as potential key regulators of Pb-induced cellular responses.

**Figure 9:**
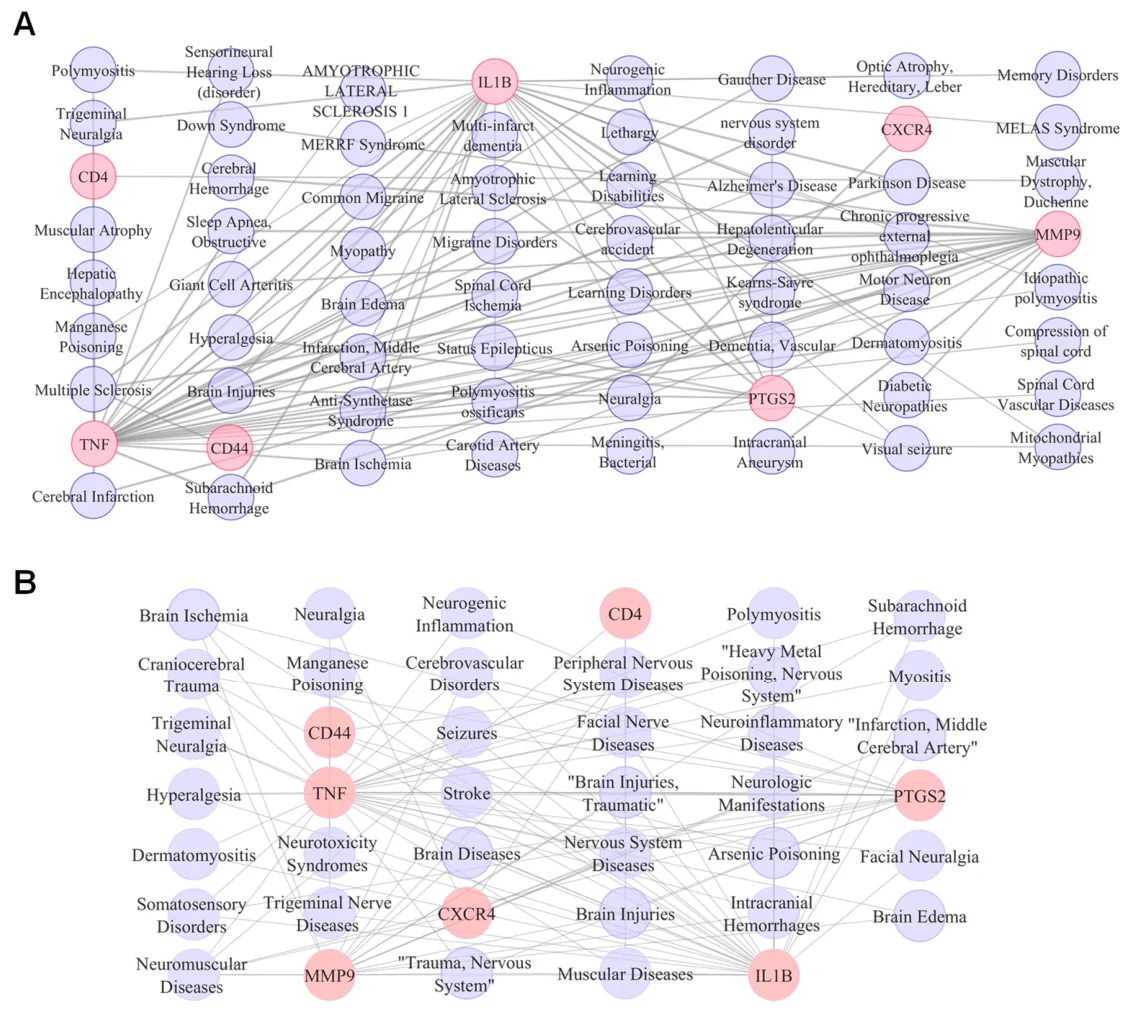
Disease association networks of hub genes in dSHSY-5Y cells treated with Pb. dSHSY-5Y cells were treated with Pb at 1.25 mM for 24 h and transcriptomic profiling was performed. (**A**) DisGeNET and (**B**) CTD gene–disease association networks of the identified hub genes (CD44, CXCR4, PTGS2, IL1B, TNF, MMP9, and CD4). Pink nodes represent hub genes, and light blue nodes represent associated disease terms.

To explore disease association networks of identified hub genes, gene–disease association networks were constructed using the DisGeNET and CTD databases to provide a systems-level view of the relationships between hub genes and nervous system-related disease phenotypes (Figure 9). The analyses revealed that hub genes (CD44, CXCR4, PTGS2, IL1B, TNF, MMP9, and CD4) were strongly associated with neurological, neurodegenerative, neurovascular, neuroinflammatory and pain-related disorders (Figure 9A,B). Among the associated conditions were Alzheimer’s disease, Parkinson’s disease, brain ischemia, cerebral infarction, stroke, neurogenic inflammation, and neuralgia (Figure 9A,B). Several associations were consistently identified across both databases and linked to neuroinflammation, neuronal injury, cerebrovascular dysfunction, and toxicant-related neurological disorders. Collectively, this network highlights the convergence of neurological and inflammatory pathways and supports the involvement of the identified hub genes in the molecular mechanisms underlying Pb-induced neurotoxicity.

## 4. Discussion

Our results show that Pb is neurotoxic to uSH-SY5Y and dSH-SY5Y cells in a concentration- and exposure duration-dependent manner. Transcriptomic profiling after toxic Pb exposure in dSH-SY5Y cells revealed the induction of genes involved in inflammatory signalling and immune-related pathways. Major hub genes were identified with links to neurological, neuroinflammatory and neurodegenerative diseases. These findings provide insight into the molecular mechanisms underlying Pb-induced neurotoxicity and highlight inflammatory pathways as potential therapeutic targets following Pb poisoning.

Cell viability data (MTT and LDH assays) showed that Pb neurotoxicity is concentration- and duration-dependent (Figure 1 and Figure 2). dSH-SY5Y cells may be more vulnerable to Pb toxicity (Figure 1 and Figure 2), which could reflect higher Pb exposure and uptake into cells. Previous studies have shown that differentiation can render SH-SY5Y cells more susceptible to toxicity from certain neurotoxicants, including alcohol and pesticides [19,20]. The IC_50_ values for Pb neurotoxicity estimated by MTT assays were approximately 2.5–3 mM after 24 h exposures, but Pb can induce loss of SH-SY5Y cell viability at a lower concentration (from 25 µM lead acetate) after a longer duration of exposure (72 h) [17]. LDH assays provided an independent method for estimating cell loss in response to Pb and yielded EC_50_ values comparable to those from MTT assays. However, for some values, these were estimates and/or predictions with broad, sometimes non-estimable, confidence intervals because the treatments did not reach 50% effect (Table 1).

Measurements of intracellular ATP levels in response to Pb exposure showed lower 24 h IC_50_ values (approximately 1 mM), indicative of disruption and/or damage of the bioenergetic machinery prior to a loss of cell viability (Figure 3). This concurs with evidence showing that Pb significantly reduces cellular ATP levels (in SH-SY5Y cells) at a concentration (5 µM) lower than that required to induce cell loss [17]. Similarly, the sensitivity of mitochondria to Pb and its impact on bioenergetics are supported by other studies [25], and we show that the induction of ROS (which are primarily liberated by mitochondria [25,26,27]) was detected at the lowest Pb concentration assessed (Figure 4). Other studies have reported Pb-induced damage to mitochondria, inhibitory effects on mitochondrial electron transport chain enzymes, and effects on antioxidant enzyme activities, all of which could contribute to the elicitation of oxidative stress as a mechanism of Pb-induced neurotoxicity [13,14,28].

Having established the conditions for inducing cell death, a sublethal concentration of Pb was selected to trigger cellular damage without complete terminal cytotoxic collapse. This concentration (1.25 mM) proved a means to study the acute molecular responses to Pb-induced neurotoxicity that arise before all cells are irreparably damaged and die. Control and Pb-treated cells had distinct transcriptomic profiles, with upregulated and downregulated DEGs (Figure 5). Combined DEG profiling, functional enrichment, and PPI network analyses provided insight into the molecular mechanisms that trigger toxicity. Significant enrichment in immune-related signalling pathways was detected via a KEGG pathway enrichment analysis of all the DEGs (Figure 5). These pathways (which were particularly common among upregulated genes) indicate an activated immune and inflammatory response. This suggests that Pb is a neuroinflammatory trigger, even in dSH-SY5Y cells that are not recognised as having inflammatory capacity. In keeping with this proposition, other studies have suggested a role for Pb as a neuroinflammatory agent [29], triggering pro-inflammatory cytokine gene expression and release from glia, including after perinatal exposures [30,31,32]. Similarly, chemokines, which act as chemoattractant cytokines, were upregulated DEGs in response to Pb exposure (Figure 5, Figure 6, Figure 7 and Figure 8), and this has also been reported for perinatal Pb exposures [30]. The initiation of transcription of immune-related markers implies that, despite their non-immune origin, dSH-SY5Y cells might initiate inflammatory-like reactions in response to toxic exposure, potentially via oxidative stress, damage-associated molecular patterns (DAMPs), and/or Toll-like receptor (TLR) activation. In support of these findings, SH-SY5Y cells can adopt a neuroimmune-like activation with the induction of inflammatory genes in response to toxic stimuli such as cyanotoxins [30]. Conversely, the downregulated DEGs were primarily associated with metabolic processes, consistent with reduced homeostatic and metabolic functions, presumably due to reduced ATP production and Pb’s ability to disrupt cellular processes through ion mimicry [7]. This split between upregulated and downregulated genes indicates a modification in cellular priorities, from preserving metabolic equilibrium to boosting an immune response.

Insights into the principal regulatory nodes within the transcriptomic landscape were derived from PPI network analysis and hub gene identification. The consistent identification of seven genes (CD44, CXCR4, PTGS2, IL1β, TNF, MMP9, and CD4) across these analytical approaches indicated their fundamental roles as molecular hubs in Pb-induced cellular responses. These genes code for proteins that typically function as mediators of immune cell recruitment and inflammation. CD44 promotes leukocyte adhesion and migration; CXCR4 codes for a chemokine receptor involved in immune responses; PTGS2 (prostaglandin-endoperoxide synthase 2) for the enzyme commonly known as cyclooxygenase-2 (COX-2), that can produce prostanoids as inflammatory mediators; IL1β and TNF are pro-inflammatory cytokines; MMP9 (matrix metalloproteinase-9) codes for an enzyme responsible for degrading extracellular matrix components, with a role in tissue remodelling and inflammatory responses; and CD4 is a co-receptor for MHC class II molecules, particularly to initiate T-cell immune responses. The induction of pro-inflammatory cytokines such as TNF-α and IL-1β has previously been associated with Pb-induced neuroinflammation in cultured cells and animal models [32,33], including increased levels in pups after maternal Pb exposure [34]. These pro-inflammatory cytokines typically increase in response to heavy metal exposure, promoting apoptotic and necrotic neuronal cell death [31,35,36].

An association of the Pb-induced candidate hub gene changes with disease highlighted conditions related to neuroinflammation, neurological disorders, and neurodegeneration (Figure 9). This is consistent with the molecular mechanisms of Pb toxicity [2,3,6,7,9,10], as well as the ascribed role and important linkage between neuroinflammation and neurodegeneration in diseases such as Alzheimer’s and Parkinson’s [37]. Consequently, changes in the expression of these candidate hub genes and their associations provide mechanistic insight into Pb exposures and poisonings, as well as their potential contribution to the pathophysiology and progression of certain neuroinflammatory and neurodegenerative diseases.

### 4.1. Study Limitations

There are limitations to our study. To assess cell viability in response to Pb exposure, a range of concentrations was explored. This approach for toxicity studies is a standard one, since it provides a means to determine the threshold of toxicity (first concentration that induces a significant reduction in cell viability) as well as the experimental or projected 50% reduction in cell viability for direct comparison with other similar (in vitro) assays, as well as context to physiologically relevant exposure concentrations. Our data suggest that neuronal cells are resistant to the neurotoxic effects of Pb, but at relatively high (supra-physiological) concentrations, a loss of cell viability is evidenced. This starting concentration for toxicity assays (0.3125 mM) (10,350 µg/dL) is notably higher than the BLL that results in encephalopathy [2,10]. However, brain tissue can accumulate and store Pb, with an estimated half-life of 2–3 years [5], thereby increasing the potential for higher concentrations and neuronal exposures in vivo. In addition, the design of our in vitro assays is to investigate the mechanism of toxicity; a process that often requires supra-physiological concentrations for acute exposures and for which the levels of the agent (Pb^2+^) taken up into cells are unknown. For Pb^2+^, this is likely to be concentration- and time-dependent and to relate to Ca^2+^ levels [5,9,38]. Furthermore, the proportion of free Pb^2+^ able to pass into cells and trigger a toxicological response will also be governed by a dynamic equilibrium between the serum albumin-bound Pb^2+^ and that which is dissociated. The serum albumin concentration could influence this in the medium used to culture uSHSY-5Y cells compared with dSHSY-5Y cells. A higher serum albumin percentage could result in lower unbound (free) Pb^2+^ relative to protein-bound Pb^2+^, depending on whether saturation thresholds have been reached, thereby affecting the relative toxicity of Pb across the different cell phenotypes.

Similarly, it is appreciated that the transcriptomic analysis was performed using a relatively high Pb exposure concentration. Concentration selection reflected an induction of cellular stress while maintaining sufficient cell viability for RNA sequencing. Furthermore, this approach enabled the investigation of early molecular responses to Pb exposure before complete cytotoxicity occurred. Nevertheless, future studies should examine lower environmentally relevant Pb exposure concentrations and durations to determine whether similar inflammatory pathways are induced. To that end, Pb exposures (up to 10 µM) have been shown to alter gene expression profiles during SH-SY5Y neuronal differentiation [39] and influence mitochondrial gene expression [40]. Secondly, the transcriptomics and bioinformatics data are informative, but further validation and quantification of specific gene expression changes are needed.

### 4.2. Summary

In summary, the transcriptomic analysis supports our molecular characterisation data, suggesting that Pb affects cellular bioenergetics and metabolism, and induces redox stress and neuroinflammation. Furthermore, the candidate transcriptomic changes are consistent with Pb’s potential to induce cognitive impairments, neurodegenerative phenotypes, and impacts on neurodevelopment. The current treatments for Pb poisoning include chelation therapy, but our pathway enrichment studies highlight the involvement of inflammatory signalling in Pb-induced neurotoxicity. Therefore, further investigation into the potential for neuroinflammation in response to Pb is warranted, as treatment with anti-inflammatories may be beneficial for individuals with Pb poisoning.

## 5. Conclusions

Pb exposure at relatively high concentrations induces marked cytotoxic and transcriptomic changes in dSH-SY5Y cells. The Pb-induced transcriptome is characterised by the activation of inflammation-related pathways and the downregulation of genes for proteins involved in cellular metabolism, neurodevelopment, and homeostasis. These findings suggest that neuronal cells can respond to Pb by inducing an inflammatory-like response, even in cells not recognised as primary drivers of inflammation. Hence, Pb may promote immunological activation at the expense of metabolic equilibrium, although further experimentation is required to replicate these findings at more physiologically relevant exposures. Furthermore, similar pathways may be activated by other cellular stresses. Nevertheless, identifying the key gene drivers for these processes provides a basis for their further characterisation and potential involvement in Pb toxicity.

## Figures and Tables

**Table 1:** Toxicity of Pb to uSH-SY5Y and dSH-SY5Y cells.

Assays	uSH-SY5Y Cells			dSH-SY5Y Cells		
	4 h	6 h	24 h	4 h	6 h	24 h
MTT (IC_50_)	6.44	5.61	3.09	5.54	4.54	2.57
95% CI	3.72–33.41	3.85–22.74	1.66–17.14	4.03–9.43	3.59–6.29	2.20–3.07
LDH (EC_50_)	2.36	2.82	4.90	2.27	2.70	3.16
95% CI	1.69–7.58	2.45–3.53	1.78–NE	NE	1.42–214.6	1.77–83.2
ATP (IC_50_)	3.45	1.76	1.38	3.43	1.64	0.93
95% CI	3.05–3.92	1.63–1.89	1.08–1.75	3.03–3.93	1.43–1.89	0.84–1.03
ROS (EC_50_)	ND	ND	2.25	ND	ND	1.26
95% CI			1.35–43.38			0.96–1.95

Toxicity parameters (IC_50_ and EC_50_ values, mM) of Pb treatment of uSH-SY5Y and dSH-SY5Y cells. ATP, adenosine triphosphate; EC_50_, half-maximal effective concentration; IC_50_, half-maximal inhibitory concentration; LDH, lactate dehydrogenase; MTT, 3-(4,5-dimethylthiazol-2-yl)-2,5-diphenyltetrazolium bromide; ND, not determined; NE, not estimable; ROS, reactive oxygen species.

## Data Availability

The original contributions presented in this study are included in the article/Supplementary Material. Further inquiries can be directed to the corresponding author(s).

## Supplementary Materials

The following supporting information can be downloaded at: https://www.mdpi.com/article/10.3390/brainsci16080889/s1, Figure S1: Production and validation of differentiated SH-SY5Y cells; Figure S2: Workflow of RNA sequencing and the bioinformatic analysis; Figure S3: Exploratory data analysis and sample clustering of RNA-seq profiles.

## Abbreviations

The following abbreviations are used in this manuscript:

ADHD	Attention-Deficit/Hyperactivity Disorder
Akt	Protein Kinase B
ANOVA	Analysis of Variance
ATP	Adenosine Triphosphate
BBB	Blood–Brain Barrier
BLL	Blood Lead Level
BLRV	Blood Lead Reference Value
BP	Biological Process
CC	Cellular Component
COX-2	Cyclooxygenase-2
CTD	Comparative Toxicogenomics Database
CXCR4	C-X-C Motif Chemokine Receptor 4
DAMPs	Damage-Associated Molecular Patterns
DCFDA	2′,7′-Dichlorofluorescein Diacetate
DEG	Differentially Expressed Gene
DESeq2	Differential Expression Sequencing 2
dSH-SY5Y	Differentiated SH-SY5Y Cells
uSH-SY5Y	Undifferentiated SH-SY5Y Cells
EC_50_	Half-Maximal Effective Concentration
ECACC	European Collection of Authenticated Cell Cultures
EMEM	Eagle’s Minimum Essential Medium
FBS	Foetal Bovine Serum
FPKM	Fragments Per Kilobase of Transcript per Million Mapped Reads
GO	Gene Ontology
HISAT2	Hierarchical Indexing for Spliced Alignment of Transcripts 2
IC_50_	Half-Maximal Inhibitory Concentration
IL1β	Interleukin-1 Beta
KEGG	Kyoto Encyclopedia of Genes and Genomes
LDH	Lactate Dehydrogenase
log_2_FC	Log_2_ Fold Change
MCC	Maximal Clique Centrality
MCODE	Molecular Complex Detection
MF	Molecular Function
MHC	Major Histocompatibility Complex
MMP9	Matrix Metalloproteinase-9
MTT	3-(4,5-Dimethylthiazol-2-yl)-2,5-Diphenyltetrazolium Bromide
NEAA	Non-Essential Amino Acid Solution
OD	Optical Density
PBS	Phosphate-Buffered Saline
PCA	Principal Component Analysis
Pb	Lead
PI3K	Phosphoinositide 3-Kinase
PPI	Protein–Protein Interaction
PTGS2	Prostaglandin-Endoperoxide Synthase 2
RA	All-trans Retinoic Acid
RNA-seq	RNA Sequencing
ROS	Reactive Oxygen Species
SEM	Standard Error of the Mean
STRING	Search Tool for the Retrieval of Interacting Genes/Proteins
TLR	Toll-Like Receptor
TNF	Tumour Necrosis Factor
