# Supplementary material for: Lead Is Toxic to Neuronal Cells by Inducing Oxidative Stress and Activating Neuroinflammatory Pathways
Source: Brain Sci. 2026 Aug 20;16(8):889. doi: 10.3390/brainsci16080889 (PMC13510954; doi:10.3390/brainsci16080889)
Supplement: Supplementary file 1 [file brainsci-16-00889-s001.zip › brainsci-4459070-supplementary.pdf]

Supplementary Material

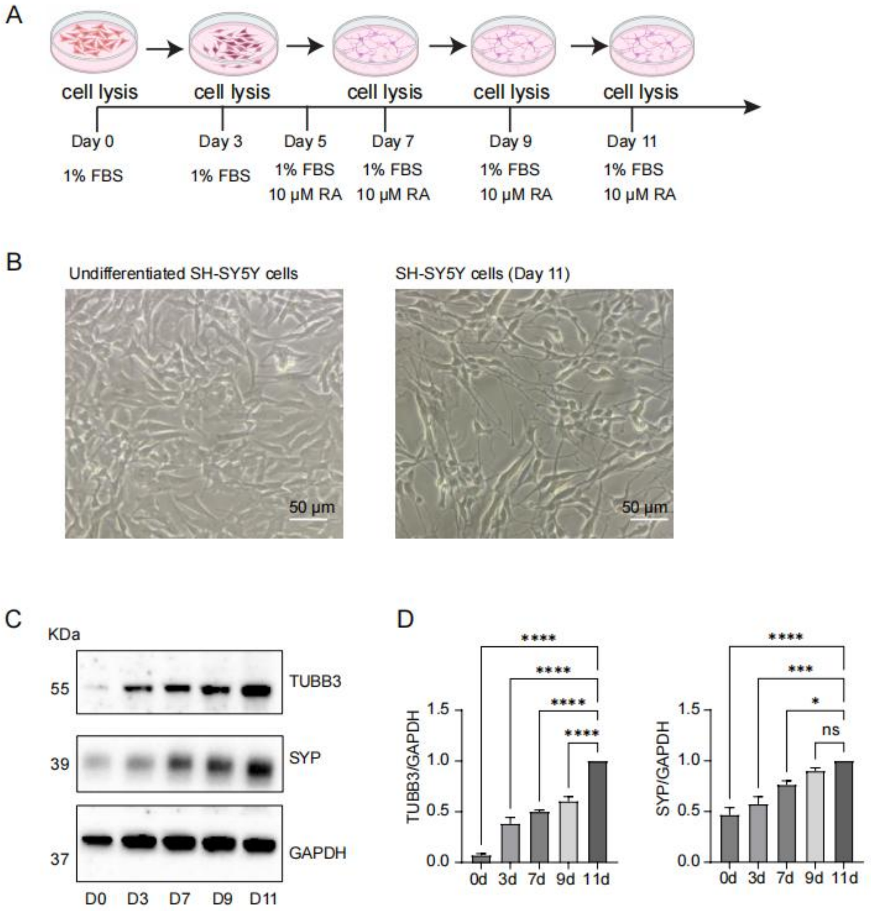

Figure S1: Production and validation of differentiated SH-SY5Y cells.

(A) Schematic illustration of the SH-SY5Y differentiation protocol. Cells were cultured in medium containing 1% FBS and differentiated with 10 μM retinoic acid (RA). (B) Representative morphological images of undifferentiated and differentiated SH-SY5Y cells (Day 11). (C) Representative western blot analysis of βIII-tubulin (TUBB3) and synaptophysin (SYP) expression during SH-SY5Y differentiation. GAPDH was used as the loading control. (D) Quantification of TUBB3 and SYP protein expression normalized to GAPDH at different days of the differentiation protocol. Data are presented as mean ± SEM. Statistical analysis was performed using one-way ANOVA. ns, not significant; *$p < 0.05$; ***$p < 0.001$; ****$p < 0.0001$. All data were from three independent experiments.

For Western blotting: cells were harvested by centrifugation at 400 × g for 5 min and lysed in RIPA buffer (Sigma, Cat#20-118) supplemented with protease inhibitor cocktail (Sigma, Cat#P3450) on ice for 30 min. Cell lysates were centrifuged at 18,000 × g for 10 min at 4°C, and the supernatants were collected. Protein samples were mixed with loading buffer, boiled for 10 min, and separated on 10% NuPAGE Bis-Tris gels

(Thermo Fisher Scientific, Cat# NP0302BOX). Proteins were transferred onto nitrocellulose (NC) membranes at 150 V for 1 h. Membranes were blocked with 1% bovine serum albumin (BSA) in PBST (PBS containing 0.05% Tween-20) and incubated overnight at 4°C with the following primary antibodies: anti-βIII-tubulin (Cell Signaling Technology, Cat# sc-365062) (1:1000), anti-synaptophysin (Proteintech, Cat# 17785-1-AP) (1:5000), and anti-GAPDH (Santa Cruz Biotechnology, Cat #5666) (1:400). After washing three times with PBST (5 min each), membranes were incubated with HRP-conjugated anti-mouse or anti-rabbit secondary antibodies (1:1000). Protein bands were visualized using an enhanced chemiluminescence (ECL) detection kit (Thermo Fisher Scientific, Cat# 32209) and imaged with a Bio-Rad ChemiDoc imaging system. Band intensities were quantified using ImageJ software.

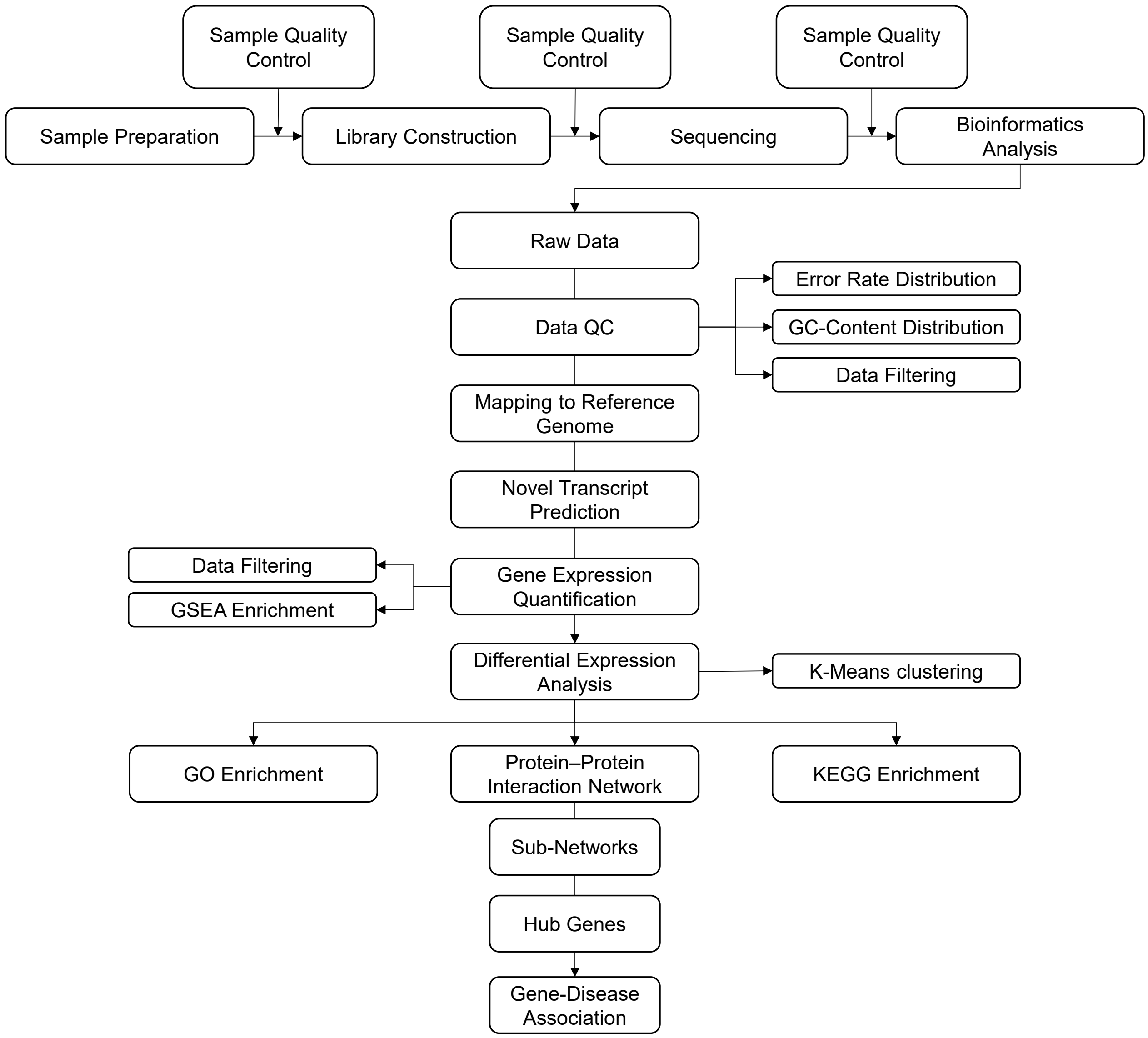

Figure S2: Workflow of RNA sequencing and the bioinformatic analysis.

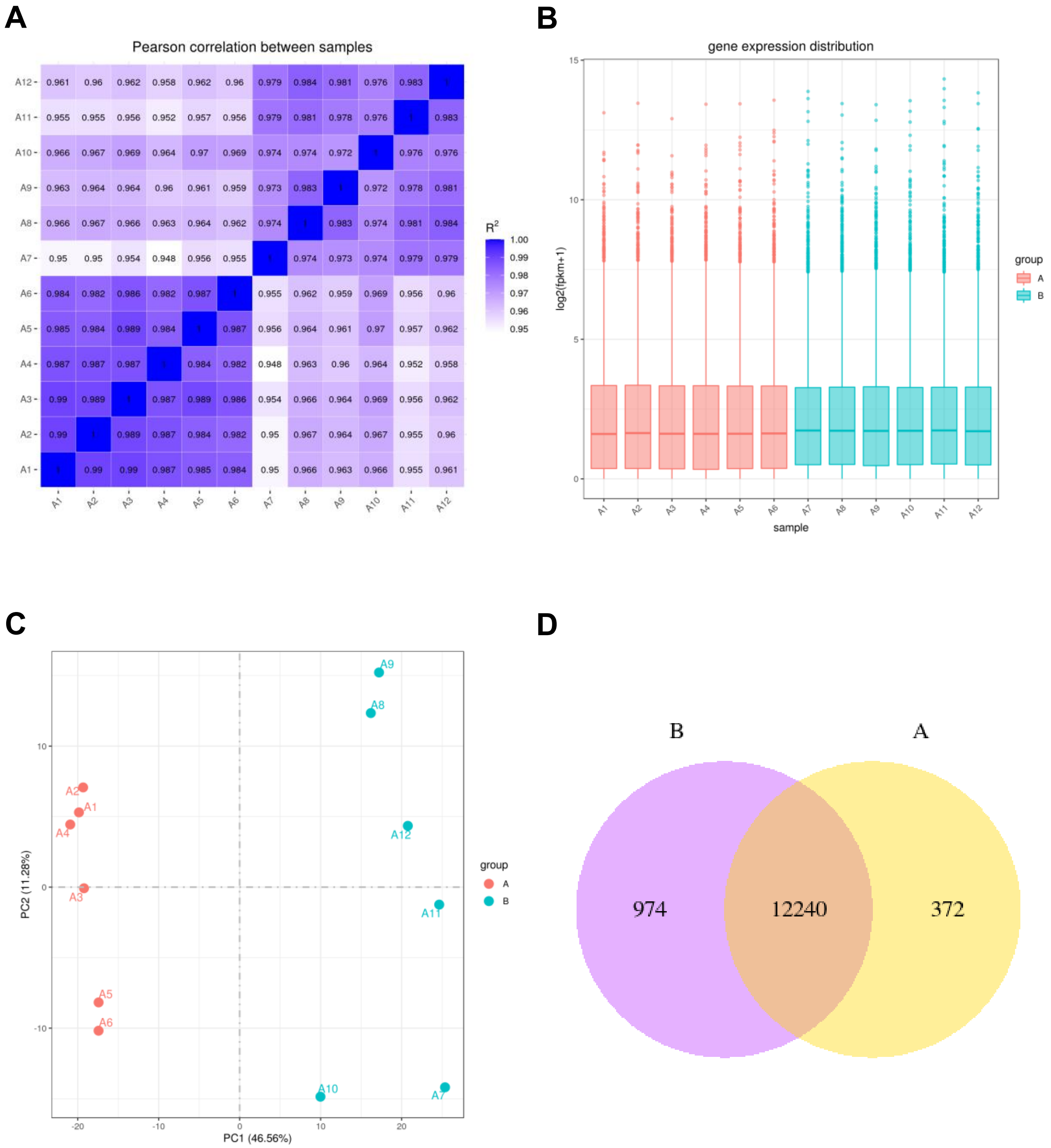

Figure S3: Exploratory data analysis and sample clustering of RNA-seq profiles.

(A) Pearson correlation matrix showing R^2 values of $\log_2$(FPKM+1) across all samples, indicating strong correlations among biological replicates ($R^2 > 0.94$). A1–A6: Pb-treated; A7–A12: controls. **(B)** Boxplot of normalized gene expression distributions across samples; treated (pink) and control (cyan) groups show consistent expression levels. **(C)** Principal component analysis (PCA) plot showing clear separation between treated and control samples, confirming distinct transcriptomic profiles. **(D)** Venn diagram depicting the number of uniquely and commonly expressed genes between Pb-treated and control SH-SY5Y cells. There were 372 genes uniquely expressed in Pb-treated cells, 974 genes specific to controls, and 12,240 genes were co-expressed in both conditions.
